# Role of macrophages in neuroimmune regulation

**DOI:** 10.3389/fimmu.2025.1573174

**Published:** 2025-06-18

**Authors:** Hairong Xiao, Kai Yang

**Affiliations:** ^1^ Department of Anesthesiology, Union Hospital, Tongji Medical College, Huazhong University of Science and Technology, Wuhan, China; ^2^ Institute of Anesthesia and Critical Care Medicine, Union Hospital, Tongji Medical College, Huazhong University of Science and Technology, Wuhan, China; ^3^ Key Laboratory of Anesthesiology and Resuscitation, Huazhong University of Science and Technology, Ministry of Education, Wuhan, China

**Keywords:** macrophages, neuroimmune, MDM, CNS, tissue-resident macrophages

## Abstract

Macrophages, as essential components of the immune system, play pivotal roles in regulating neuroimmune interactions. These cells exist in two primary forms: tissue-resident macrophages and monocyte-derived macrophages (MDMs), each exhibiting distinct functions in neuroimmune responses. Tissue-resident macrophages maintain tissue homeostasis and act as the first line of defense against pathogens, while MDMs are recruited during inflammation, performing both phagocytic and immunomodulatory functions. Despite the recognized roles of these macrophage populations in systemic immunity, their specific contributions to neuroimmune regulation remain poorly understood. This review aims to elucidate the differential roles of tissue-resident macrophages and MDMs in the neuroimmune pathway. We explore their mechanisms of activation, interaction with other immune cells, and involvement in inflammatory processes within the central nervous system (CNS). By identifying the distinct and overlapping functions of these macrophage populations, this review may provide novel insights into therapeutic strategies aimed at modulating neuroimmune responses, particularly in the context of neuroinflammatory disorders such as neurodegenerative diseases.

## 1 Introduction

Macrophages are versatile immune cells that play critical roles in maintaining tissue integrity, initiating immune responses, and resolving inflammation (1, 2). While macrophages were historically categorized into M1 (pro-inflammatory) and M2 (anti-inflammatory) states, recent studies reveal a spectrum of phenotypes shaped by ontogeny (3), metabolic rewiring (4), and epigenetic reprogramming (5). Tissue-resident and monocyte-derived macrophages exhibit distinct transcriptional profiles and functional plasticity, challenging the traditional polarization paradigm (6). In the context of neuroimmune regulation, macrophages, particularly microglia (the resident macrophages of the brain), are critical in maintaining homeostasis within the central nervous system (CNS) and modulating neuroinflammation (1, 2). However, the precise contributions of tissue-resident macrophages and MDMs to neuroimmune responses are not fully understood. Existing studies have demonstrated that tissue-resident macrophages, such as microglia, play a central role in neuroinflammation, yet the functional differences between tissue-resident macrophages and MDMs in the brain’s immune responses remain unclear. Neuroimmune regulation encompasses bidirectional communication between the nervous and immune systems, where neural signals (e.g., neurotransmitters, neuropeptides) modulate immune cell activity, and immune-derived molecules (e.g., cytokines) influence neuronal function. Macrophages are pivotal in this axis, expressing receptors for neurotransmitters (e.g., α7nAChR, β2-adrenergic receptors) and releasing immunomodulatory factors that shape neuroinflammatory responses. In the CNS, this involves microglia (resident macrophages) and infiltrating monocyte-derived macrophages, which collectively maintain homeostasis, respond to injury, and contribute to pathologies like Alzheimer’s disease. This review focuses on how these macrophage populations differentially integrate neural and immune signals to regulate neuroinflammation (7). Additionally, while BMDMs have been shown to migrate to the CNS during injury or infection, their role in modulating neuroimmune pathways has not been systematically examined (4, 8).

Building on this foundation, it is crucial to explore further the nuanced interactions of macrophages within the CNS, especially in the context of prevalent neurological disorders. Diseases such as Alzheimer’s disease, Parkinson’s disease, and multiple sclerosis are marked by distinct patterns of neuroinflammation, where microglia and infiltrating MDMs play potentially divergent but critical roles. For instance, in Alzheimer’s disease, microglia are implicated in the clearance of amyloid-beta plaques, a hallmark of the disease pathology. Understanding these dynamics is vital for developing targeted interventions that could modulate macrophage activity to halt or reverse the progression of these debilitating diseases (9, 10).

This review aims to fill this gap by investigating the specific roles of tissue-resident macrophages and MDMs in neuroimmune regulation. By examining their activation, recruitment, and interaction with other immune cells within the neuroinflammatory milieu, we aim to provide a comprehensive understanding of how these two macrophage populations contribute to neuroimmune responses. This review could offer insights into new therapeutic strategies for modulating macrophage function in neuroinflammatory diseases, thereby opening avenues for potential treatments in conditions such as Alzheimer’s disease, multiple sclerosis, and traumatic brain injury. Clinical evidence supports these mechanistic insights. Single-cell RNA sequencing of human CNS macrophages reveals conserved transcriptional programs with murine models (e.g., TREM2+ microglia in Alzheimer’s), while vagus nerve stimulation trials in rheumatoid arthritis patients demonstrate reduced TNF-α levels, mirroring murine anti-inflammatory reflexes (6, 10). These parallels underscore the translational relevance of macrophage-centric therapies.

## 2 Macrophages

Macrophages are versatile innate immune cells that perform several core physiological functions essential for tissue homeostasis and defense. These include phagocytosis of pathogens, cellular debris, and apoptotic cells; production of cytokines and chemokines that modulate immune and neural responses; antigen processing and presentation to T cells via major histocompatibility complex molecules; and orchestration of tissue repair and re therapeutic potential of targeting this triad modeling through secretion of growth factors and extracellular matrix components (9, 11). These canonical roles provide a foundation for their critical involvement in neuroimmune crosstalk, where macrophages contribute to maintaining neural health and resolving inflammation.

Emerging evidence reveals that macrophage phenotypes extend beyond the binary M1/M2 model, reflecting complex niche-specific adaptations as detailed below.

### 2.1 Beyond M1/M2: niche-driven macrophage heterogeneity

The classical M1/M2 dichotomy, while useful for conceptualizing macrophage activation *in vitro*, fails to capture the phenotypic diversity and functional plasticity of macrophages *in vivo*. Recent advances reveal that macrophage behavior is dynamically shaped by tissue-specific niches, where ontogeny, local microenvironmental cues, and metabolic-epigenetic cross-talk drive a spectrum of activation states beyond binary classifications (4, 6). For instance, tissue-resident macrophages (TRMs) derived from embryonic precursors exhibit distinct transcriptional and epigenetic programs compared to monocyte-derived macrophages (MDMs), enabling them to maintain homeostasis or respond to injury in a tissue-tailored manner (10). Meanwhile, local factors—such as cytokines (e.g., TGF-β, IL-10), metabolites (e.g., itaconate, succinate), and neural inputs—orchestrate transient yet context-specific phenotypes that defy M1/M2 labels (4, 12). Single-cell technologies further uncover spatial heterogeneity, with macrophages co-expressing ‘M1’ (e.g., Nos2) and ‘M2’ (e.g., Arg1) markers simultaneously in inflamed tissues (13). This paradigm shift underscores the need to study macrophages through the lens of their niche, where ontogeny, metabolism, and spatial positioning collectively determine their role in neuroimmune regulation. Murine MDMs exhibit further heterogeneity, with subsets such as Ly6C+ inflammatory monocytes differentiating into CCR2+ macrophages during neuroinflammation, while Ly6C- subsets contribute to tissue repair. These distinctions are critical for understanding their divergent roles in CNS pathologies (4, 10).

### 2.2 Function of resident and monocyte-derived macrophages

Tissue-resident macrophages (TRMs) and MDMs exhibit fundamental differences in their ontogeny, metabolic programming, and functional specialization. TRMs originate from embryonic precursors (yolk sac/fetal liver) and maintain tissue-specific identities through unique epigenetic signatures (e.g., H3K4me1 marks at enhancers), enabling long-term homeostatic functions independent of bone marrow contributions (4). Their distinct developmental origin is coupled with a metabolic preference for oxidative phosphorylation (OXPHOS), which sustains anti-inflammatory functions and tissue repair capacities (12).

In contrast, MDMs are derived from hematopoietic stem cells and adopt transient, inflammation-driven phenotypes characterized by glycolytic metabolism and mitochondrial ROS (mtROS) production (4, 12). This metabolic reprogramming drives NLRP3 inflammasome activation in MDMs, equipping them for potent pro-inflammatory responses during pathogen clearance. Spatial mapping studies reveal how this metabolic zonation plays out in disease contexts - for example, mtROS+ BMDMs preferentially localize to necrotic cores in atherosclerotic plaques, while OXPHOS-dependent TRMs dominate peripheral regions (4).

Within tissues, TRMs serve as specialized sentinels - alveolar macrophages in lungs, microglia in CNS, and Kupffer cells in liver - maintaining homeostasis through their OXPHOS-mediated anti-inflammatory programs (4). When activated, they recruit circulating monocytes that differentiate into BMDMs, which then employ their glycolytic-mtROS axis for intensive phagocytosis and cytokine production (14, 15). This partnership is evident in neuroinflammation, where microglia (TRMs) initiate responses through OXPHOS-dependent mechanisms, while recruited BMDMs drive NLRP3-mediated inflammation through mtROS (4).

The tissue microenvironment ultimately constrains this functional specialization. Even when MDMs repopulate tissues (as in aging cochlea where they replace embryonic-derived TRMs (16), they adopt tissue-appropriate metabolic profiles. However, under pathological conditions like chronic inflammation, this metabolic flexibility can break down, leading to dysfunctional macrophage responses that exacerbate disease (4).

### 2.3 Factors that differ tissue-resident macrophage from MDMs

Tissue-resident macrophages primarily exhibit anti-inflammatory or inflammation-suppressing functions, whereas MDMs display mainly proinflammatory responses. For example, both are activated in cerebral hemorrhage cases and participate in phagocytosis and tissue repair; however, compared with tissue-resident macrophages, MDMs exhibit superior antigen-presenting and phagocytic abilities (14).

What causes these differences in their immunoregulatory functions? First, tissue-resident and bone marrow-derived macrophages have different expression profiles. In lung injury models, recruited monocytes/macrophages can cause bilateral lung tissue damage, whereas tissue-resident macrophages play additional anti-inflammatory and repair roles. Moreover, alveolar macrophages possess more apoptotic cell recognition receptors than circulating monocytes and dendritic cells (17).

Additionally, these two types differ in their immunometabolism. Since resident alveolar macrophages do not rely on glycolysis during lipopolysaccharide (LPS)-induced inflammation, the low glucose content in the microenvironment might inhibit the production of proinflammatory factors to initiate inflammatory responses (18). Tissue-resident immune cells also impact systemic metabolism (19, 20). For example, tissue-resident macrophages show strong insulin sensitivity (21), whereas liver Kupffer cells regulate lipid metabolism (22).

Furthermore, the function of tissue-resident macrophages is influenced by the severity (23), duration, and location of inflammation. In local inflammatory responses, alveolar macrophages control the progression of inflammation and tissue repair by regulating the extent of tissue damage (23). During acute inflammatory responses that impact the entire body, the tissue-resident macrophage concentration increases and may affect the entire system, such as myocardial ischemia and sepsis. Furthermore, alveolar macrophages undergo prime activation during inflammation and strengthen the defense of the lung against infections (24). Subcutaneous Bacillus Calmette–Guerin (BCG) injection can induce lung memory macrophage formation via the brain-gut axis (25), thereby demonstrating the role of remote inflammation in influencing macrophage responses in other tissues. As previously mentioned, tissue macrophages in other body parts are activated during acute inflammatory responses, enhancing local immune defense. However, epigenetic changes in alveolar macrophages can lead to long-term lung immune paralysis after chronic inflammation (26).

The location of tissue-resident macrophages also affects their role in the inflammatory response. In intestinal mucosal immunity, lamina propria macrophages facilitate inflammation progression and epithelial barrier damage, whereas muscularis macrophages, located deeper in the intestinal wall, exhibit anti-inflammatory and tissue-protective functions (27). Adipose tissue macrophages (ATMs) exemplify niche-specific specialization. In obesity, ATMs adopt a proinflammatory phenotype (CD11c+), impairing insulin sensitivity and releasing leptin, which disrupts blood-brain barrier integrity. Conversely, lean-state ATMs (CD206+) enhance insulin signaling via IL-10 secretion and may migrate to the hypothalamus, linking metabolic dysfunction to neuroinflammation (28, 29). In lung tissue, alveolar and interstitial macrophages also present some differences that stem from spatial and pathogenic signal associations. On the one hand, different modes of gene expression regulation are present; on the other hand, cell-specific remote regulatory factors are present in locally accessible chromatin. For example, reduced Toll-like receptor 4 (TLR4) ligand expression in alveolar macrophages also decreases LPS stimulation-induced inflammatory responses (30).

As previously mentioned, tissue-resident macrophages (TRMs) may originate from MDMs. Jenkins et al. (31) reviewed the parameters that regulate tissue-resident macrophages in addition to the environment and reported that long-lived TRMs not only compete with newcomers but also reverse their identity or function, similar to MDMs. Bleriot et al. (32) summarized the roles of tissue-resident macrophages in four areas: ontogeny and perspective, local factors unique to the residence niche, inflammation status, and macrophage adaptation. This framework provides a valid basis for conversion, which offers new therapeutic approaches for resident macrophage-related diseases, such as targeted alveolar macrophage treatment in hereditary pulmonary surfactant protein deposition disease through third-generation, self-inactivating lentivirus engineering (33). Additionally, interactions between different tissue macrophages may lead to tissue-resident macrophage migration. For example, adipose tissue macrophages migrating to the hypothalamus cause microglial proliferation in obese mice (34).

## 3 Macrophages in neuroimmune regulation

The nervous system involves three classical regulatory pathways: the HPA axis (hypothalamic-pituitary-adrenal axis), the sympathetic nervous system, and the parasympathetic nervous system. Their activation modes, resulting effects (inflammatory or anti-inflammatory responses), and pathway interconnections are both independent and interrelated. Their interconnection is demonstrated by direct or indirect HPA axis activation postsympathetic or parasympathetic nervous system activation. This releases cortisol, affects β2-adrenergic receptor expression on immune cells, and synergizes with the sympathetic nervous system to exert anti-inflammatory effects (35). The activation of the vagus nerve’s afferent branch by electrical acupuncture stimulation via the NTS (nucleus tractus solitarius) releases adrenal hormones that suppress inflammatory responses in the adrenal glands (36). In rats with vagus nerve dysfunction, the peripheral and central nervous systems can partially influence inflammatory responses (37), indicating the diversity of neuroimmune pathways. Falvey et al. also reported that direct activation of the paraventricular nucleus of the hypothalamus after electrical stimulation of the carotid sinus nerve in mice activates the HPA axis, thereby activating macrophage glucocorticoid receptors to alleviate inflammatory responses (38).

In addition to the adrenal glands, nerve fibers also directly innervate immune organs (39). However, neurotransmitters have a short circulatory half-life and face difficulty in activating a sufficient number of immune cells. Therefore, on the one hand, neurotransmitters such as acetylcholine are amplified through lymphoid tissues, thereby affecting immune cell function in nearby and distant immune organs (40). The role of the nervous system in immune cell migration is particularly evident in the significant impact of the sympathetic nervous system on leukocyte migration (41). However, this complex process correlates with leukocyte adhesion molecule alterations, chemokine receptor expression, and endothelial cell responsiveness to corresponding receptors, circadian rhythms, inflammation, and sympathetic nervous system activation status, as well as the patient’s overall health. Additionally, cholinergic signaling and sensory neurons also help regulate leukocyte migration.

**Figure 1:**
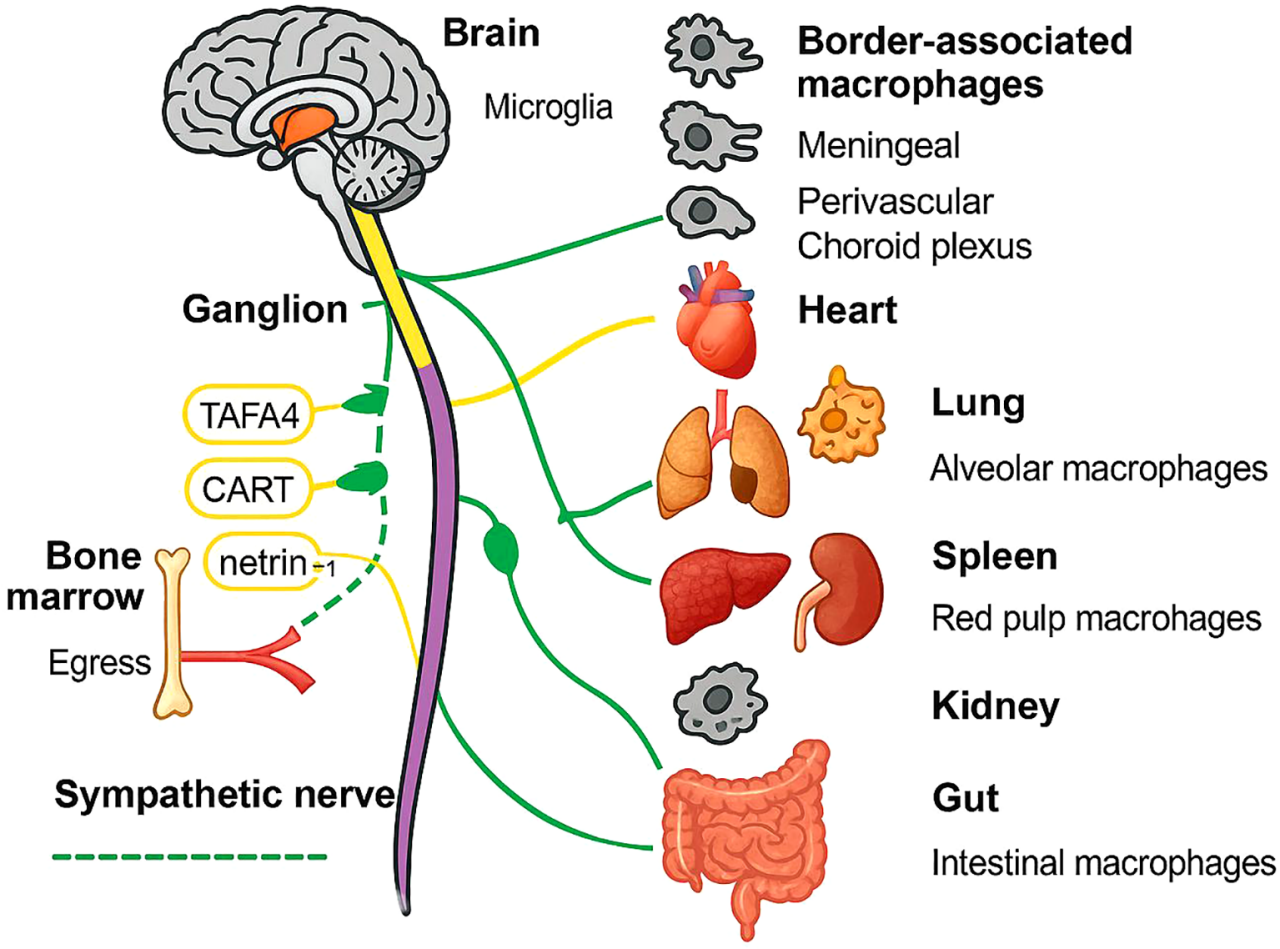
Schematic overview of autonomic neuro-immune communication across major organs. Solid yellow and dashed green lines depict the vagus and sympathetic nerve trunks, respectively, emanating from the brain through a representative ganglion to peripheral targets. Monocyte egress from the bone marrow is shown in red, with netrin-1 facilitating mobilization. Tissue-resident macrophages are illustrated for each organ: microglia and border-associated macrophages (meningeal, perivascular, choroid plexus) in the CNS; alveolar macrophages in lung; red pulp macrophages in spleen; renal macrophages in kidney; intestinal macrophages in gut. Key neuroimmune mediators (TAFA4, CART, netrin-1) are annotated along their respective nerve fibers.

On the other hand, nerve fibers are distributed close to macrophages (**Figure 1**), although their interactions are still unknown. While synaptic-like structures akin to neuromuscular junctions have not been verified to date, it has been suggested that macrophages express receptors for a few neurotransmitters on their surface, such as cholinergic (especially alpha nicotinic acetylcholine receptor (nAChR)) and adrenergic receptors.

In addition to neurotransmitter secretion, neurons can also secrete various immune-regulating proteins for immune regulation. Moreover, macrophages also express a few receptors for these immune-regulating proteins on their surface. Various techniques, such as high-resolution imaging, viral tracing, single-cell transcriptomics, and optogenetics, have confirmed the presence of such specific sensory neurons with immune-regulatory functions in the lymph nodes (42). These sensory neurons can directly or indirectly regulate macrophage function through immune-regulating proteins and participate in immune regulation. For example, TAFA4 enhances macrophage tissue repair function (43), and cocaine- and amphetamine-regulated transcript preprotein (CART protein) are released and regulate the immune cell functions of the spleen (44). The netrin-1 protein mediates the anti-inflammatory effects of the vagus nerve (45). Transient receptor potential ankyrin 1 (TRPA1) proteins moderate IL-1β-induced vagal afferent fiber activation (46), and colony-stimulating factor 1 (CSF1) proteins help maintain and develop alveolar macrophages (47). Macrophage migration inhibitory factor (MIF) not only possesses glucocorticoid-antagonist properties but also has proinflammatory and pathogenic roles (48). However, no studies have reported whether the abundance of neurotransmitters and immune-regulating protein-related receptors differs between tissue-resident macrophages and MDMs.

Recent discoveries have further illuminated the complex role of macrophages in neuroimmune regulation. Specialized LYVE1+ meningeal macrophages act as sentinels at CNS borders, recruiting circulating monocytes via CCL2-dependent trafficking during neuroinflammation, with real-time imaging revealing their dynamic breach of the blood-brain barrier (49). This recruitment pathway operates alongside the classical neural regulatory systems, while under hypoxic conditions characteristic of brain injury or tumors, macrophages employ metabolic adaptations like itaconate production to suppress succinate dehydrogenase and modulate inflammatory cytokine release (4). These mechanisms complement the known neuroimmune pathways involving the HPA axis, sympathetic/parasympathetic systems, and neurotransmitter signaling, demonstrating how macrophages integrate neural, metabolic, and cellular cues to shape neuroinflammatory outcomes.

## 4 Microglia and astrocytes in neuroimmune crosstalk

Microglia and astrocytes are critical partners in neuroimmune regulation. Microglia, the CNS-resident macrophages, rapidly respond to injury via TREM2-dependent phagocytosis and NLRP3 inflammasome activation, while astrocytes modulate MDM recruitment through CCL2 and TGF-β secretion (49). In Alzheimer’s disease, microglial dysfunction impairs amyloid-beta clearance, exacerbating MDM-driven inflammation. Conversely, astrocyte-derived IL-33 polarizes MDMs toward an anti-inflammatory phenotype, highlighting the therapeutic potential of targeting this triad (29).

In addition to microglia, the central nervous system hosts distinct populations of border-associated macrophages located in the meninges, perivascular spaces, and choroid plexus. These macrophages originate from both yolk sac and monocyte-derived precursors and are renewed dynamically, maintaining their populations independently of microglia (6, 49). Functionally and anatomically distinct from parenchymal microglia and infiltrating monocytes, these border-associated macrophages play critical roles in CNS immune surveillance, antigen drainage, and neurovascular regulation. For example, meningeal macrophages facilitate the sampling of cerebrospinal fluid antigens and coordinate T cell recruitment, while perivascular macrophages modulate blood-brain barrier permeability and immune cell trafficking. Choroid plexus macrophages contribute to immune monitoring within the ventricular system and participate in cerebrospinal fluid homeostasis (29). These specialized macrophage subsets form an essential interface between the CNS and peripheral immune system, modulating neuroimmune communication and impacting the pathogenesis of neurological diseases.

## 5 Vagus nerve and macrophages

Vagus nerve activation primarily exerts anti-inflammatory effects. Low vagus nerve activity levels were shown to be responsible for increased all-cause mortality in two large population studies (50). Prolonged endogenous acetylcholine deficiency exacerbates the inflammatory response in mice with pulmonary edema (50).

Vagal afferent fibers also contain TLRs, which directly sense signals from DAMPs and other receptors (44) after inflammation. Tsaava et al. suggested that specific vagus nerve stimulation parameters can modulate plasma cytokine levels in the absence of inflammation and contribute to homeostasis maintenance (51). However, in chronic inflammatory conditions such as high-fat diet-induced inflammation, cholinergic receptor expression decreases, resulting in immune paralysis and extensive inflammatory damage (52). However, in CLP (cecal ligation and puncture) sepsis survivor mice subjected to a secondary LPS challenge, high vagus nerve activity mediates immune paralysis and exacerbates host immune injury (52). Vagus nerve stimulation can also cause side effects such as bradycardia. Therefore, the specificity and timing of vagus nerve activation can provide a new direction for future research in electrostimulation therapy. Recent studies on function-specific neural fiber structures and neurons of the vagus nerve have provided a structural theoretical basis for additional tissue- or function-specific vagus nerve stimulation research, such as calcium imaging and nerve analysis (53). Clinically, fascicular vagus nerve stimulation has now been shown to selectively activate splenic macrophages to reduce systemic inflammation while avoiding cardiac side effects (6), validating these anatomical findings. Therefore, neural fibers and macrophages present more opportunities for further exploration.

Upon activation, the vagus nerve releases acetylcholine, and cholinergic receptors are activated on central or peripheral macrophages. This enhances the release of anti-inflammatory factors and reduces the release of proinflammatory factors, thereby exerting anti-inflammatory and pro-resolution effects (7). Additionally, the vagus nerve, by releasing immunoregulatory proteins, modulates immune cell functions. Furthermore, it also facilitates cellular interactions, such as those in cholinergic neurons, that regulate calcium ion currents in endothelial cells for intestinal injury repair.

As a critical immune organ, the spleen hosts various immune cells that mobilize different immune cells and perform various immunoregulatory functions during inflammation (54). However, the role of the spleen in the cholinergic anti-inflammatory reflex is ambiguous (55). In the myocardial ischemia-induced inflammatory response, high-Ly6C monocytes migrate to the inflammation site for immune responses, whereas spleen macrophages do not migrate to the inflammation site. Specific pro-resolving mediators (SPMs) secreted by macrophages can significantly resolve myocardial inflammation (56). In the cholinergic neural reflex, spleen macrophages release fewer proinflammatory factors and reduce systemic inflammatory responses (54). Vagus nerve activation inhibits α7nAChR+CD11b+ granulocyte migration from the spleen to damaged lung tissue induced by LPS, thereby alleviating lung injury (56). Disruptions in macrophage phenotypic balance—as shaped by their tissue microenvironment and metabolic cues—contribute to pathologies such as multiple sclerosis (10). Vagus nerve stimulation skews this balance by upregulating M2 markers (e.g., Arg1, IL-10) in MDMs, suggesting a pathway to mitigate neuroinflammation (4). Nonetheless, this study could not explain the activation of the cholinergic receptors of lung tissue macrophages.

Nonimmune organs, such as the lungs and lung macrophages, exert anti-inflammatory effects, which have been confirmed to alleviate proinflammatory factors (57) and promote pro-resolving factors. Yang et al. also proposed an independent pulmonary parasympathetic reflex that possesses structures and functions relevant to neuroimmune reflexes (58). Additionally, Jayaprakash et al. distinguished the specificity of innervating different organs and functions within vagus nerve fibers on the basis of quantitative immunohistochemistry, myelination, and other tissue characteristics and selectively activated the vagus nerve bundles to perform specific functions (53).

Post-vagus nerve activation, α7nACh receptor activation occurs in tissue-resident macrophages in the intestinal muscle layer (non-spleen-derived macrophages), which results in reduced expression of MPO and inflammatory factors (55). Furthermore, α7nACh receptor activation in peritoneal macrophages minimizes the occurrence of postoperative enteritis (59). Additionally, α4β2 cholinergic receptor activation in lamina propria macrophages of the small intestine enhances their ability to phagocytose bacteria (60). Remarkably, gut macrophages can also migrate retrogradely along the vagus nerve to the brain in Parkinson’s disease models, potentially transporting α-synuclein aggregates and directly linking intestinal inflammation to neurodegeneration (28).

## 6 Sympathetic nervous system and macrophages

The role of the sympathetic nervous system in immune regulation is uncertain and appears to be more influenced by the surrounding microenvironment. Functional neuroimmune interactions depend on the microenvironment in experimental settings, where after noninfectious factors are induced, splenic SND (sympathetic nerve discharge) and splenic-derived cytokine expression are directly recorded under physiological stress. Notably, α2ARs (α2-adrenergic receptors) inhibit IL-6 secretion in a sterile environment, whereas adrenaline release inhibits IL-6 through β2ARs (β2-adrenergic receptors) in the presence of bacteria (35). A study on the immune regulation of arthritis by the sympathetic nervous system revealed that adrenergic receptor activation depends on the distance from the adrenaline source: catecholamines in the vicinity preferentially activate β receptors and exert anti-inflammatory effects, whereas catecholamines at a distance activate α receptors, thereby exerting proinflammatory effects (61).

In local tissues, sympathetic nervous system activation, in turn, activates adrenergic receptors on local macrophages, thereby exerting anti-inflammatory effects. For example, in inflammatory reactions, α2 receptor blockade and β2 receptor activation in lung macrophages have protective effects on the lungs (62). Additionally, adrenaline signaling in muscular layer macrophages mitigates intestinal inflammation-induced neuronal damage (28), whereas optical activation of the colonic sympathetic nervous system reduces colitis by limiting immune cell extravasation (63). Recent work reveals a gut-brain-sympathetic axis where intestinal IFN-γ+ macrophages activate enteric neurons to drive CNS inflammation in EAE (multiple sclerosis models), with blockade of gut macrophage-derived IFN-γ ameliorating disease (29). Moreover, local sympathectomy enhances anti-inflammatory responses and reduces paclitaxel-induced mechanical and cold allodynia in mice (64). Additionally, interstitial lung macrophages are intimately associated with TH-positive sympathetic nerve fibers and can alleviate lung inflammation (47).

The sympathetic nervous system can significantly impact immune cell migration in neuroimmune pathways (65). Human studies corroborate these mechanisms: in multiple sclerosis patients, sympathetic dysfunction correlates with increased CNS macrophage infiltration, while clinical trials of β2-adrenergic agonists (e.g., salbutamol) demonstrate reduced neuroinflammation, mirroring murine data (6). Single-cell analyses further reveal conserved transcriptional programs between human and mouse macrophages, validating translational relevance. The differences in local sympathetic nervous system activity are caused by circadian rhythms driven by neural and humoral signals. These variations in adhesion molecules induce diurnal differences in immune cell trafficking from the bone marrow into the circulation. Furthermore, sympathetic nervous system activation also causes peripheral vasoconstriction, decreased tissue oxygen supply, and increased intercellular calcium ion concentrations, thereby reducing cell migration independent of adrenergic receptors. Additionally, the degree and duration of inflammation can impact the regulation of immune cell migration.

## 7 Conclusion

As active participants in tissue homeostasis and immune defense, macrophages are jointly regulated by neural and hormonal factors. Tissue-resident macrophages maintain tissue homeostasis under neural regulation by exerting local anti-inflammatory and pro-resolving effects. They also affect the diffusion of pro-resolving factors both locally and in the bloodstream, in addition to reducing the concentration of proinflammatory factors in local tissues. However, MDMs are influenced by various neural (sympathetic, vagus, and sensory neurons) and hormonal factors. They participate in the trafficking of substances from the bone marrow, lymphoid organs, and tissues under various stimuli, such as day-night cycles, inflammation, stress, and tumors. This phenomenon modulates the body’s immune response, which can either exacerbate or suppress inflammation.

In summary, this complexity may be greatly influenced by the degree and duration of inflammation, as well as the proximity of the inflammation site. The transmission of inflammatory signals through the nervous system’s afferent end can modulate the response of effector immune cells at the efferent end. In both the vagus nerve and the sympathetic nervous system, tissue-resident macrophages, which maintain tissue homeostasis, still primarily exhibit inflammation-resolving functions. The role of migrating macrophages is more complicated, and the vagus nerve seems to alleviate proinflammatory effects and promote anti-inflammatory effects. The sympathetic nervous system can significantly impact immune cell migration in the bone marrow. Therefore, more studies are needed on selective neural activation, including particular neural fibers, organs, and cells, in the field of neuroimmune regulation.
